# *Trichoderma* Enhances Net Photosynthesis, Water Use Efficiency, and Growth of Wheat (*Triticum aestivum* L.) under Salt Stress

**DOI:** 10.3390/microorganisms8101565

**Published:** 2020-10-11

**Authors:** Abraham Mulu Oljira, Tabassum Hussain, Tatoba R. Waghmode, Huicheng Zhao, Hongyong Sun, Xiaojing Liu, Xinzhen Wang, Binbin Liu

**Affiliations:** 1 Center for Agricultural Resources Research, Key Laboratory of Agricultural Water Resources, Institute of Genetics and Developmental Biology, Chinese Academy of Sciences, Shijiazhuang 050021, China; abrahammulu@yahoo.com (A.M.O.); thussain@uok.edu.pk (T.H.); tatobawaghmode@yahoo.com (T.R.W.); hczhao@sjziam.ac.cn (H.Z.); hysun@sjziam.ac.cn (H.S.); xjliu@sjziam.ac.cn (X.L.); xzwang@sjziam.ac.cn (X.W.); 2 University of Chinese Academy of Sciences, Beijing 100039, China; 3 Institute of Sustainable Halophyte Utilization, University of Karachi, Karachi 75270, Pakistan

**Keywords:** *Trichoderma*, *Bacillus*, wheat, IAA, salt stress

## Abstract

Soil salinity is one of the most important abiotic stresses limiting plant growth and productivity. The breeding of salt-tolerant wheat cultivars has substantially relieved the adverse effects of salt stress. Complementing these cultivars with growth-promoting microbes has the potential to stimulate and further enhance their salt tolerance. In this study, two fungal isolates, Th4 and Th6, and one bacterial isolate, C7, were isolated. The phylogenetic analyses suggested that these isolates were closely related to *Trichoderma yunnanense*, *Trichoderma afroharzianum*, and *Bacillus licheniformis*, respectively. These isolates produced indole-3-acetic acid (IAA) under salt stress (200 mM). The abilities of these isolates to enhance salt tolerance were investigated by seed coatings on salt-sensitive and salt-tolerant wheat cultivars. Salt stress (S), cultivar (C), and microbial treatment (M) significantly affected water use efficiency. The interaction effect of M x S significantly correlated with all photosynthetic parameters investigated. Treatments with *Trichoderma* isolates enhanced net photosynthesis, water use efficiency and biomass production. Principal component analysis revealed that the influences of microbial isolates on the photosynthetic parameters of the different wheat cultivars differed substantially. This study illustrated that *Trichoderma* isolates enhance the growth of wheat under salt stress and demonstrated the potential of using these isolates as plant biostimulants.

## 1. Introduction

Soil salinization is recognized as one of the most serious threats to agricultural production [1], affecting more than one billion hectares worldwide [2]. Increased salinization is expected to affect 50% of arable land by the year 2050 [3]. Previous studies have revealed the detrimental effect of salt stress on many aspects of plant gas exchange, including net photosynthesis, transpiration rates, intercellular CO_2_, stomatal conductance, and water use efficiency [4,5]. The enhancement of photosynthetic gas exchange efficiency under stressful environmental conditions is critical for yield improvement [6]. In particular, the improvement of net photosynthesis and water use efficiency has attracted researchers’ attention as possible approaches to optimize carbon assimilation in food crop production [7,8]. Metabolic acclimation via the synthesis of compatible solutes, including proline, has shown the potential to minimize salt-induced oxidative stress, protect the integrity of photosynthetic machinery (thylakoids and plasma membranes), repair damage to photosystem II, and scavenge reactive oxygen species (ROS) [9,10].

Wheat is a major cereal crop in the North China Plain, and given that salinity stress has major effects on crop productivity in this area [11,12], greater attention has been given to breeding salt-tolerant cultivars over the last few decades [13]. Major advancements have been made in developing cultivars resilient to environmental stress, and evidence is still emerging for the contribution of plant-microbe interactions to the further improvement of this resistance [14,15,16]. The application of plant growth-promoting microbes (PGPM) has been shown to overcome problems related to salinity [17,18]. Certain *Trichoderma* and *Bacillus* spp. have effectively induced plant tolerance to salt stress by modulating several traits in wheat and other crops. *Trichoderma longibrachiatum* T6 enhanced wheat seedling tolerance to salinity, presumably through improvement of the antioxidative defense system and scavenging of excessive ROS produced by plants under salt stress [19,20]. *Trichoderma harzianum* improved the uptake of beneficial elements, stimulated compatible solute accumulation, and elevated the level of antioxidant enzymes in *Brassica juncea* L. under salt stress [21]. Similarly, *Bacillus licheniformis* HSW-16 ameliorated the negative effects of NaCl stress on wheat growth [22], and *Bacillus subtilis* BERA 71 inoculation improved biomass and photosynthetic pigments and reduced ROS in chickpea under saline soil conditions [23].

Plants cope with salt stress by employing several strategies, such as proline accumulation in the chloroplast and cytoplasm to maintain an external osmotic balance is one potential strategy [24,25]. A study by Hayat et al., 2012 demonstrated that optimum proline accumulation in the plant had a positive correlation with high tolerance to environmental stress [26]. However, there is contradictory information on whether the proline content of seedlings increases or decreases during plant-microbe interactions under abiotic stress. A review of the literature by Chun et al., 2018 about proline accumulation under salinity stress in plants colonized by arbuscular mycorrhizal fungi (AMF) concluded that AMF inoculation increases, decreases, or has no effect on proline accumulation in the plants [27]. Furthermore, proline is a source of carbon and nitrogen for fungi when nutrients are limited, and this may be a key survival strategy under stressful conditions [27].

Microbial seed coating is an attractive approach to counter salt stress even though it is utilized infrequently in crop protection against abiotic stresses [28]. There is a growing interest in applying seed coating technology in precision agriculture due to its convenience, ecological safety, and economic advantages [29]. Several reports have shown that seed inoculation with microbes capable of indole-3-acetic acid (IAA) secretion efficiently improved plant tolerance to salt stress [30,31,32]. In addition, many cultivable salt-tolerant microbial strains isolated from saline environments survive under osmotic and ionic stresses and are known to mitigate the detrimental effects induced by a variety of abiotic stresses [33,34].

Studies have shown the prospective advantages of seed coating with PGPM for enhancing the productivity of crops under low-input agriculture [35,36]; however, little information is available regarding wheat seed coating with IAA producing beneficial microbes and whether the effects can ameliorate the deleterious effects of salt stress. Salt stress inhibits the production of IAA in the roots and restricts lateral root development [37,38]. In a prior study, IAA-secreting fungal species, such as *Trichoderma*, promoted *Arabidopsis* growth under normal and salt stress conditions [39,40]. Seed treatment with *Bacillus* spp. has the potential to induce physiological changes and protect plants under adverse environmental conditions [41].

The interaction between plants and beneficial soil microbes, including *Trichoderma* and *Bacillus*, can have a large impact on plant tolerance to salt stress. Thus, we hypothesized that the PGPM that are capable of producing IAA can promote plant growth under salt stress and enhance salt tolerance, and these effects could be reflected by altered proline accumulation and photosynthetic activity. Insights into cultivar-specific microbe interaction responses could lead to the selection of efficient isolates and open up a new avenue of research for practical applications. The objective of this study was (a) to isolate *Trichoderma* spp. from high saline soil and *Bacillus* from pig farms, (b) to determine the potential of these isolates for secretion of IAA under salt stress, and (c) to investigate the influence of microbial seed treatment containing these isolates on leaf proline content and photosynthetic parameters.

## 2. Materials and Methods

### 2.1. Microbial Isolation and Purification

The soil sample used for the isolation of halotolerant *Trichoderma*, herein referred to as “Th4 and Th6” was collected from the rhizosphere of a *Tamarix chinensis* Lour in a long-term phytoremediation field experimental site in Haixing County, Hebei Province, China. The soil chemical and physical properties were described in our recently published study [42]. The inoculums were isolated following serial dilution on *Trichoderma* Selective Media (TSM) [43]. Briefly, serial dilutions were made by suspending 0.3 g of soil in 29.7 mL of sterile distilled water. The mixture was vortexed vigorously in a 50 mL Falcon tube for 10 min. After serial dilution, 100 µL of the 10^3^–10^5^ suspensions were pipetted onto Petri plates containing TSM, and the plates were incubated at 25 °C. After four days, *Trichoderma* colonies were collected with a sterile spatula and transferred to fresh Czapek Dox Agar (Himedia) for purification. Likewise, *Bacillus* C7 was obtained from composted manure from a pig farm located in Nanpi County, Hebei Province, China. Pig manure has been used as an organic amendment of coastal saline soil for the production of wheat [44]. Following serial dilution, 100 µL of the 10^3^–10^5^ dilutions were pipetted on carboxymethyl cellulose sodium salt (pH 7.0) agar medium and incubated at 50 °C. A beaker filled with sterilized water was placed in the incubator containing the plates to prevent the medium from drying out. Pure inoculums were obtained by picking single colonies of the isolate aseptically using a sterile inoculation loop on a clean bench.

### 2.2. DNA Extraction, Phylogenetic Marker Gene Amplification, and Sequencing

Genomic DNA of the fungal isolates was extracted from the pure isolates using an E.Z.N.A.^®^ High-Performance Fungal DNA Kit (Omega Bio-tek, Inc., Norcross, GA, USA). The amplification of the fungal internal transcribed spacer (ITS) gene was performed using primers ITS1 (5′TCCGTAGGTGAACCTGCGG-3′) and ITS4 (5′TCCTCCGCTTCTTGATTGATATGC-3′) [45]. PCR was carried out in 50 µl reaction volumes containing 5 μL of 10 × EasyTaq^®^ Buffer with 1 μL of EasyTaq^®^ DNA polymerase, 4 μL of 2.5 mM dNTPs, 1 μL of each primer (TransGen Biotech Co., Ltd., Beijing, China), 2 μL of template DNA, and 38 μL of sterile double distilled water in a thermal cycler (Bio-Rad). The reaction involved initial denaturation at 95 °C for 5 min, followed by 30 cycles of denaturation at 94 °C for 40 sec, annealing at 58 °C for 40 sec, and extension at 72 °C for 4 sec, with a final extension step at 72 °C for 5 min [46].

Bacterial genomic DNA was extracted from a pure culture using the TIANamp Bacteria DNA Kit (TIANGEN, Biotech, Beijing Co., Ltd., Beijing, China) according to the manufacturer’s protocol. PCR was conducted in 50 µL reaction volumes containing 25 μl of 2×GoldStar Best Master mix (TaKaRa, Biotechnology, Dalian, China), 0.3 μL of each primer (TransGen Biotech Co., Ltd., Beijing, China), 2 μL of template DNA, and 22.4 μL of sterile double distilled water. The 16S rRNA gene was amplified using the primer pair 27F (5′-AGAGTTGATCCTGGTCAG-3′) and 1492R (5′- GGTTACCTTGTTACGCTT-3′) [47]. The reaction involved initial denaturation at 95 °C for 5 min, followed by 29 cycles of denaturation at 95 °C for 1 min, annealing at 55 °C for 1 min, and extension at 72 °C for 2 min, with a final extension step at 72 °C for 10 min.

The PCR products were sequenced at Beijing (Sangon Biotech Co., Ltd., Shanghai, China). The nearest neighbors of each sequence were obtained through a BLASTN search of the GenBank nucleotide database [48]. Phylogenetic trees were constructed for the isolates with the neighbor-joining method using MEGA X software [49].

### 2.3. Microbial Inoculum Preparation

*Trichoderma* spore powder and *Bacillus* cell pellets were prepared according to methods described previously [50,51]. In brief, *Trichoderma* was grown on boiled wheat grain for three weeks, and the conidial masses were separated in a 50 mL Falcon tube by centrifugation at 9500× *g* for 10 min. The pelleted conidia were collected and dried under aerated and aseptic conditions for three days. The dried mass was powdered with a High-Speed Universal Disintegrator lab grinder (Model No. FW80, Huanghua Faithful Instrument Co. Ltd., China). The *Bacillus* isolate was incubated in 100 mL of LB broth media for 24 h at 30 °C on a shaker at a speed of 150 rpm (ZWY-200D, Shanghai ZHICHENG Analytical Instruments Manufacturing Co., Ltd., Shanghai, China). Approximately 30 mL of culture was transferred to a sterilized 50 mL Falcon tube and centrifuged at 4 °C and 9500× *g* for 10 min. The supernatant was discarded, and the cell pellet was washed three times with sterilized distilled water to remove media residue.

### 2.4. Determination of Indole-3- Acetic acid Secretion

For the determination of IAA secretion from the Th4 and Th6 isolates, 2 mL of spore suspension (inoculum 10^8^ spore mL^−1^) was grown in a 250 mL flask containing 200 mL Czapek Dox broth [30] amended with 0.5 gl-1 tryptophan and incubated at 27 °C with shaking at 150 rpm for six days. Similarly, 2 mL of C7 inoculum (OD = 0.6) was transferred to LB broth and incubated at 27 °C with shaking at 150 rpm for 24 h. The media were amended with NaCl concentrations of 0 mM NaCl and 200 mM NaCl. After the incubation period, the biomass was removed by centrifugation for 5 min at 10,000× *g* to collect the supernatant. The IAA concentration in the culture supernatant was assessed with Salkowski reagent, and the reaction was allowed to proceed for 20 min in the dark [52]. The absorbance was measured at 530 nm using a UV spectrophotometer (SHIMADZU, UV-2450, Dongguan HongCheng Optical Products Co. Ltd., Dongguan, China).

### 2.5. Seed Treatment and Seedling Growth Conditions

Two winter wheat cultivars with contrasting levels of salt stress tolerance were selected: Shimai and Xiaoyan60, herein referred to as “salt-sensitive” and “salt-tolerant”, respectively. The cultivars were obtained from the Plant Genetics and Breeding Research Section, Center for Agricultural Resource Research (CARR). Xiaoyan60 was developed for tolerance to biotic and abiotic stresses [13]. Shimai is among the wheat cultivars widely cultivated in the North China Plain [53]. The seeds were surface sterilized in 70% ethanol for 10 min and 1% NaOCl for 2 min and then washed four times with sterilized distilled water. Dry powder of the *Trichoderma* isolates was mixed with sterilized 1.5% xanthan gum gel following the procedure described previously [54,55]. We used a *Trichoderma* spore powder for seed coating that was easy to handle and enhanced the practical applicability at the farmer level. The seeds were coated with Th4 and Th6 at a rate of 10 g of fungal powder per kg of wheat seeds [56]. The *Bacillus licheniformis* C7 cell pellet was dissolved in approximately 30 mL of sterilized distilled water (OD660 = 0.6), and the suspension was added to sterilized 1.5% xanthan gum gel in a beaker. The inocula and the seeds were gently stirred into the xanthan gum mixture for 10 min using a stirring rod. The wet seeds were removed from the beaker and dried for 3 h on an aerated clean bench under aseptic conditions. In the control (without microbial coating), seeds were immersed in 1.5% sterilized xanthan gum gel only with a similar procedure as that used for coating the seeds. The coated seeds were placed onto two layers of moistened filter paper in Petri plates. After one week, 30 (15 for each cultivar) uniformly emerged wheat seedlings were transferred to hydroponic vessels containing Hoagland solution under aerated conditions [57]. The seedlings were grown hydroponically for 14 days and exposed to either 0 mM NaCl or 200 mM NaCl. In the 200 mM NaCl treatment, 4.38 g of NaCl was gradually added to the Hoagland nutrient solution once every two days to a final concentration of 200 mM NaCl, whereas the same amount of Hoagland solution without NaCl was added to the 0 mM NaCl-treated vessel. The growth room conditions were as follows: 21 °C, 16-h photoperiod, 400 µmol m^−2^ s^−1^ light intensity, and 55% relative humidity. In this study, root colonization was confirmed following the described method in [58], with some modifications. Briefly, the root surfaces were sterilized in 1% NaOCl for 2 min followed by rinsing with sterile distilled water. The root tissue was homogenized in sterilized mortar and pistil under aseptic conditions. The root homogenates belonging to the *Trichoderma* treatment were serially diluted and plated onto TSM, whereas those belonging to the *Bacillus* treatment were plated onto Luria-Bertani (LB) medium.

To evaluate the influence of the microbial seed treatment on the growth of wheat seedlings, the plant biomass weight, proline contents and photosynthetic parameters were measured after 11 days of seedling growth under salt stress treatment. The experiment was conducted for 32 days after sowing (seven days in Petri plates, 14 days in hydroponic, and 11 days in hydroponic conditions with 0 mM NaCl or 200 mM NaCl).

### 2.6. Determination of Proline Contents

The proline content was determined by using fully expanded leaves according to a method reported previously [59]. Briefly, freshly harvested leaves (0.5 g) of six plants per treatment were homogenized with 6 mL of 3% aqueous sulfosalicylic acid. After centrifugation, 2 mL of supernatant was mixed with 2 mL of ninhydrin reagent and 2 mL of glacial acetic acid, and then incubated in a 100 °C boiling water bath for 1 h. The proline content was measured at 520 nm using a UV-spectrophotometer (SHIMADZU, UV-2450) after extraction with 4 mL of toluene for 20 min. Then, the proline content was determined using the calibration curve and expressed as µmole proline g^−1^ fresh weight (FW).

### 2.7. Photosynthetic Parameter Measurements

Gas exchange measurements were conducted on the fully expanded third leaves (three plants) from each treatment using an LI-6400XT portable photosynthesis measurement system (Li-Cor Biosciences, Lincoln, NE, USA). Net photosynthesis (*A*: μmol CO_2_ m^−2^ s^−1^), stomatal conductance (*g_s_*: mol H_2_O m^−2^ s^−1^), intercellular CO_2_ (*C_i_*: μmol CO_2_ m^−2^ s^−1^), and transpiration rates (*E*: mmol H_2_O m^−2^ s^−1^) were measured at a photosynthetically active radiation (PAR) of 500 µmol photon m^−2^ s^−1^, 400 µmol CO_2_ m^−2^ s^−1^, with a 400 µmol s^−1^ flow rate, at 40-60% humidity, and 27 ± 2.5 °C. The water use efficiency (*WUE* = A/E, µmol CO_2_ mmol^−1^ H_2_O) was also calculated.

### 2.8. Statistical Analysis

An analysis of variance (ANOVA) was performed, followed by comparisons of the least squares means. F-tests were employed to evaluate the effects of cultivar (C), microbial treatment (M), salt stress (S), and their interactions. The above analysis was conducted with R software version 3.5 using the ‘Car’ [60] and ‘lsmeans’ [61] packages. All results given are means ± standard errors (SE). Principal component analysis (PCA) was employed to verify the variation and compare the patterns induced as a result of the treatments.

## 3. Results

### 3.1. Phylogenetic Analyses and Determination of Indole-3-Acetic Acid (IAA) Secretion

In the current study, a total of eight *Trichoderma* isolates from the *Tamarix chinensis* rhizosphere and 12 *Bacillus* isolates from pig manure were isolated and screened for their IAA secretion abilities. Three IAA-producing strains (Th4, Th6 and C7) were selected for further analysis. The C7 isolate secreted a significantly (*p* < 0.05) higher amount of IAA under 200 mM NaCl treatment than under 0 mM treatment. Compared with C7, fungal isolates Th4 and Th6 produced dramatically lower levels of IAA (Table 1).

**Figure 1:**
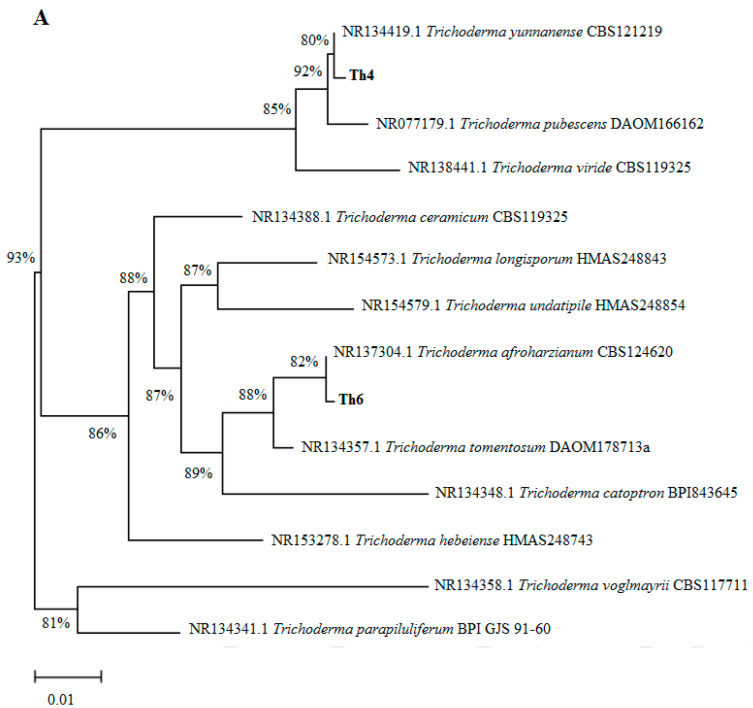

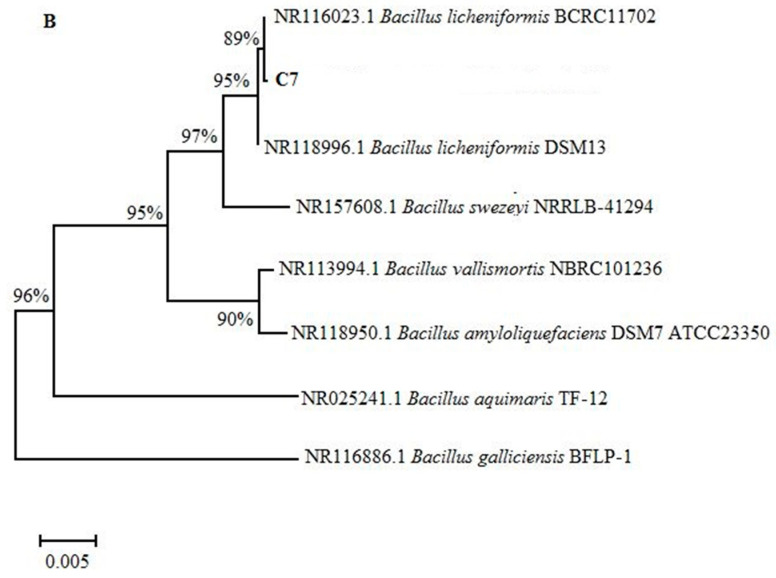
Phylogenetic tree showing the relationship among (**A**) Th4 and Th6 based on sequencing results of the ITS region and (**B**) C7 based on sequence results of the 16S rRNA gene.

Fungal and bacterial isolates were identified by sequencing their internal transcribed spacer (ITS) and 16S rRNA genes, respectively. The Th4, Th6 and C7 isolate nucleotide sequences were subjected to basic alignment local tool (BLAST) analysis against the National Center for Biotechnology (NCBI) database. Phylogenetic analysis based on ITS showed that the Th4 and Th6 isolates had a close relationship with *Trichoderma yunnanense* and *Trichoderma afroharzianum*, respectively (Figure 1A). Likewise, the phylogenetic analysis based on 16S rRNA showed that the C7 isolate showed the highest similarity with *Bacillus licheniformis* (Figure 1B). The nucleotide sequences of Th4, Th6, and C7 isolates have been deposited in the NCBI under accession numbers MT762351.1, MT762352.1, and MT762371.1, respectively.

### 3.2. Proline Contents

The effects of cultivar (C), microbial seed treatment (M), and salt stress (S) on the proline content were assessed (Table 2). Under unstressed conditions (0 mM NaCl), no significant difference in the proline content was noted between the uncoated and the three seed-coating treatments of cultivar Shimai, while a significant decrease in proline contents with the seed-coating treatments was observed for Xiaoyan60. In the treatments with salt stress (200 mM NaCl), a significantly lower proline content was observed in the three seed-coating treatments than in the uncoated treatment of cultivar Shimai; for cultivar Xiaoyan60, the C7 coating treatment increased the proline content to a level higher than that of the uncoated treatment, while the Th4 and Th6 coating treatments yielded lower proline contents than the uncoated treatment. The three factors (C, M, and S) all showed significant effects on the proline content (Table 2B).

### 3.3. Photosynthetic Parameters

**Figure 2:**
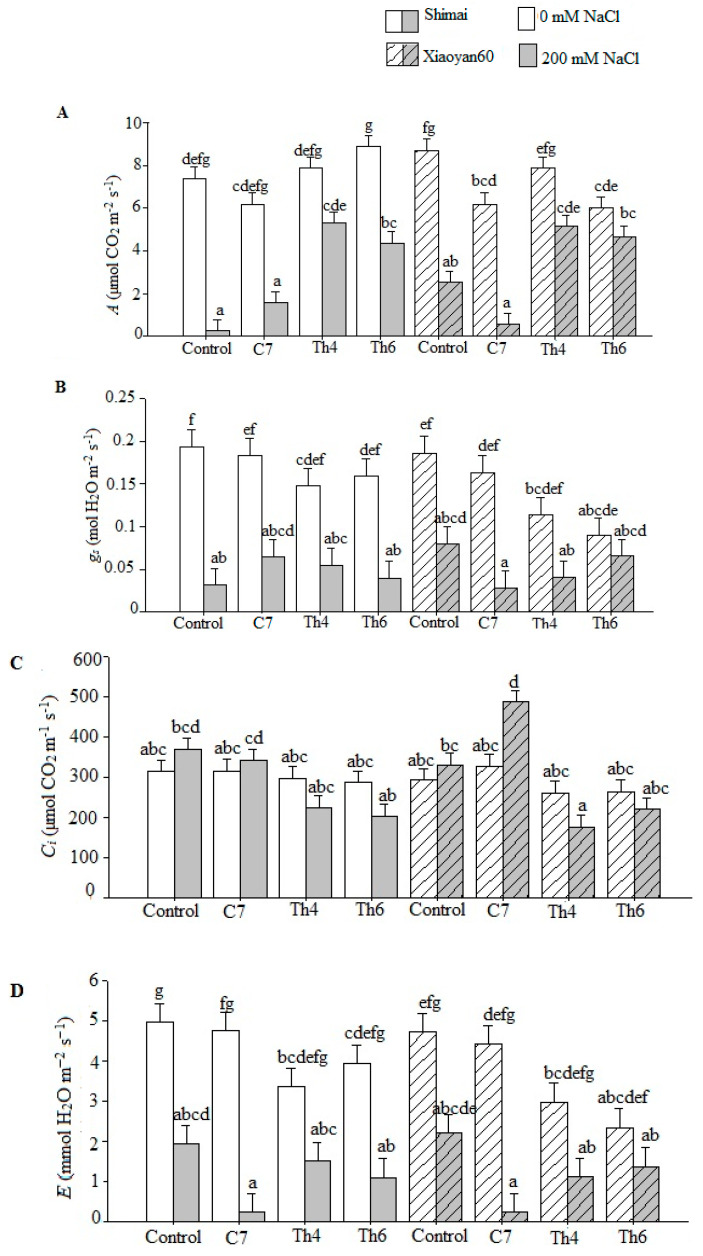

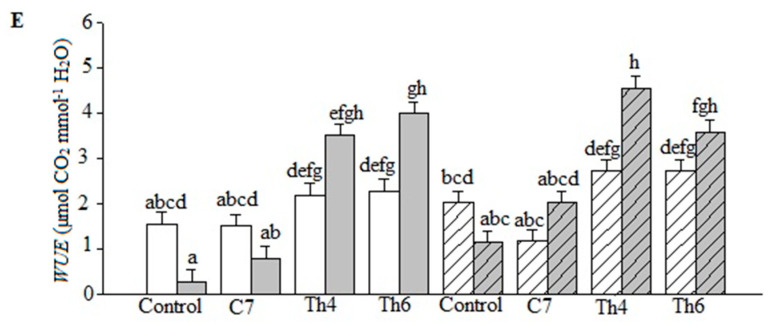
Photosynthetic parameters of wheat cultivars Shimai and Xiaoyan60 under the microbe and salt stress treatments. Control, C7, Th4, and Th6 represent uncoated seeds, C7 seed coating, Th4 seed coating, and Th6 seed coating, respectively. The salt stress levels were 0 mM NaCl and 200 mM NaCl. The values are the means of three replicates ± SE (*n* = 3). (**A**) Net photosynthesis (*A*), (**B**) stomatal conductance (*g_s_*), (**C**) intercellular CO_2_ (C*_i_*), (**D**) transpiration (*E*), and (**E**) water use efficiency (*WUE*). The wheat cultivars: Shimai in white-gray bars and Xiaoyan60 in white-gray bars with course pattern. Different letters denote significant differences between the treatments and the cultivars according to Tukey-adjusted comparisons (*p* < 0.05). Means sharing a letter are not significantly different.

Photosynthetic parameters, including net photosynthesis (*A*), stomatal conductance (*g_s_*), intercellular CO_2_ (*C_i_*), transpiration (*E*) and water use efficiency (*WUE*), were measured to understand the influence of cultivar, microbial seed treatment, and salt stress on photosynthetic parameters in wheat seedlings. The net photosynthesis (*A*) with the control and C7 treatments decreased under salt stress treatment. However, seedlings of both cultivars had relatively elevated *A* when the seeds were coated with Th4 and Th6 (Figure 2A). Further analysis revealed that *A* was significantly (*p* < 0.05) correlated with salt stress, microbial strain, and their interactive effects but not with the interactive effects of all three main factors (C × S × M) (Table 3). The influence of salt stress on stomatal conductance (*g_s_*) was significant (*p* < 0.001, Table 3). Under nonstress conditions, seedlings grown from coated seeds exhibited nonsignificant lower stomatal conductance than control plants. Except for the interaction between microbial seed treatment x salt stress (M × S), none of the interactions of the main effects were significant (Table 3).

The influence of seed treatment with different microbial strains (M) on intercellular CO_2_ (*Ci*) was significant (*p* < 0.001, Table 3). The *Ci* was higher in the control and C7-coated seedlings than in the Th4 and Th6 seedlings (Figure 2C) regardless of cultivar and salt stress exposure. Microbial seed treatment × salt stress (M × S) and cultivar × microbial seed treatment (C × M) interactions were significantly correlated with intercellular CO_2_ (*p* < 0.001; *p* < 0.05, respectively, Table 3).

Both salt stress and microbial strain significantly (*p* < 0.05) influenced the leaf transpiration rate (*E*). The seedlings grown from Th4 and Th6 exhibited consistently lower E than control plants irrespective of cultivar or salt stress (Figure 2D). The microbial seed treatment x salt stress (M x S) interaction was significantly (*p* < 0.01) different for *E* (Table 3).

The leaf *WUE* was significantly (*p* < 0.01) correlated with all main factors (cultivar, microbial strain, and salt stress), with an interactive effect of microbial strains and salt stress (Table 3). Under stress conditions, *WUE* was found to be higher in seedlings grown from Th4 and Th6 irrespective of cultivar (Figure 2E).

**Figure 3:**
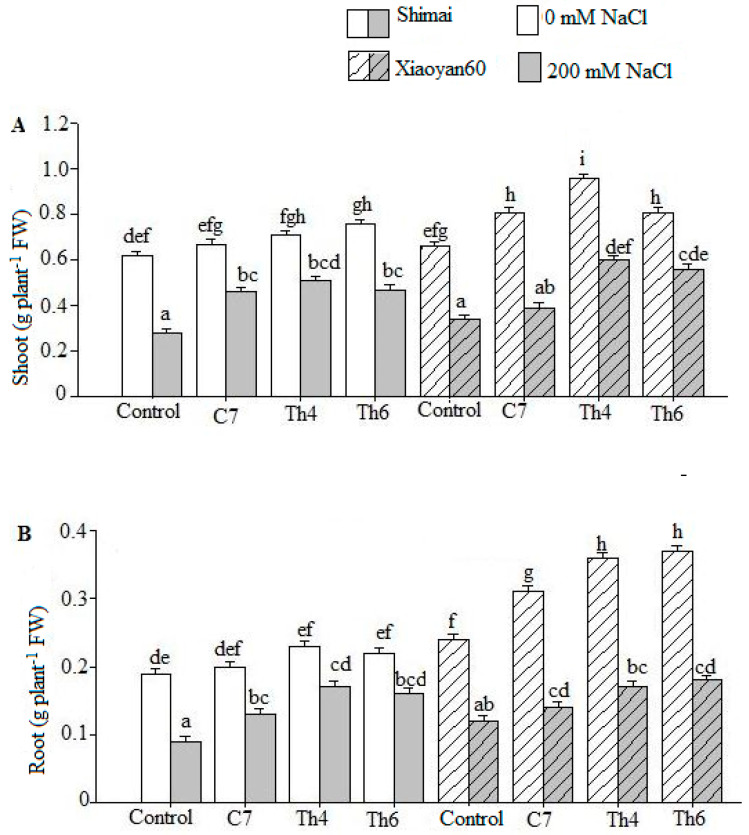
Biomass production of Shimai and Xiaoyan60 under microbe and salt stress treatments. Control, C7, Th4, and Th6 represent uncoated seeds, C7 seed coating, Th4 seed coating, and Th6 seed coating, respectively. The plant was harvested from the hydroponic vessel at the end of the experiment (32 days after sowing). The shoot (**A**) and root (**B**) fresh samples were wiped dry using soft paper towel, and the fresh weight was recorded immediately. The values are the means of three replicates ± SE, (*n* = 3). The wheat cultivars: Shimai in white-gray bars and Xiaoyan60 in white-gray bars with course pattern. Different letters denote significant differences between the treatments and the cultivars according to Tukey-adjusted comparisons (*p* < 0.05). Means sharing a letter are not significantly different.

Under unstressed conditions, microbial isolates increased the seedling biomass of both cultivars compared to the control treatment. Salt stress treatment severely reduced the biomass; however, microbial seed treatment, particularly *Trichoderma* isolates, improved the shoot and root biomass weight (Figure 3 and Table 4).

### 3.4. Principal Component Analysis of Cultivar-Specific Photosynthetic Parameters

**Figure 4:**
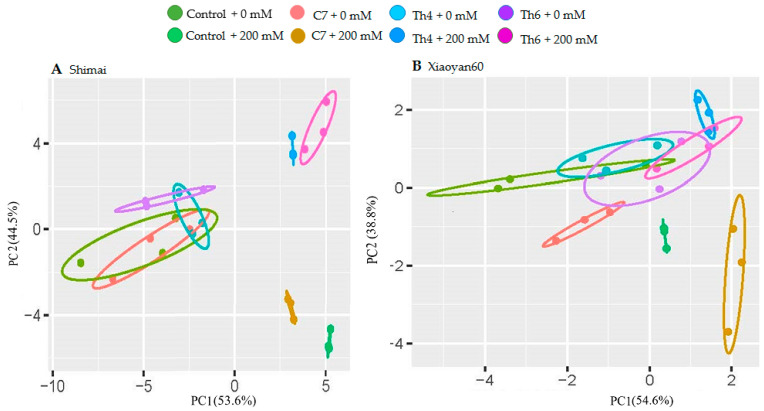
Results of two-dimensional principal component analysis (PCA) for photosynthetic gas exchange parameters in two wheat cultivars: (**A**) Shimai and (**B**) Xiaoyan60. Control + 0 mM and control + 200 mM are combinations of uncoated seed with 0 and 200 mM NaCl, respectively. C7 + 0 mM, Th4 + 0 mM, Th6 + 0 mM, C7 + 200 mM, Th4 +200 mM and Th6 +200 mM are combinations of seed coating treatments with C7, Th4, and Th6 under 0 and 200 mM NaCl, respectively.

Figure 4 shows the PCA of the photosynthetic parameters (*A*, *g_s_*, *C_i_*, *E*, and *WUE*) for both cultivars. PC1 and PC2 accounted for 53.6% and 44.5% of the total variation, respectively, for Shimai (Figure 4A). A similar pattern of total variation (54.6% and 38.8%) was explained in Xiaoyan60 by PCA1 and PC2 (Figure 4B), respectively. In the treatments without salt stress, limited separation was exhibited between seedlings with seed coating treatments and control plants. In the salt stress treatments, seedlings were separated by the microbial strain used for seed treatment for both cultivars except that an overlap was noted for Xiaoyan60 coated with Th6 and Th4. In general, microbial seed coating and salt stress caused strong variation in the photosynthetic parameters in both studied cultivars.

## 4. Discussion

Maintaining endogenous concentrations of phytohormones using the seed priming technique was shown to have a positive impact on salt stress resistance in wheat [62,63]. The utilization of PGPM to enhance wheat tolerance against salt stress has been considered an important eco-sustainable approach [15]. *Bacillus* produced high amounts of IAA compared to *Trichoderma* isolates. However, the production of high IAA was not correlated with plant growth in a *Bacillus* treatment. When used in seed treatment, beneficial root-colonizing microbes that secrete IAA can potentially manipulate the plant’s endogenous pool of IAA, contributing to host tolerance to the harmful effects of environmental stress [64,65]. However, the amount of microbial IAA required by the host may vary within an extremely narrow range based on plant phenotype; as a result, the plant-microbe interaction outcomes are sometimes diverse and highly dependent on the respective species [66,67]. Comprehensive studies are necessary to identify the level of IAA required for plant growth in a specific microenvironment.

In previous studies, increased accumulation of proline in leaf, root, and stem tissues was reported as a common response to salt stress in Jerusalem artichoke [68] and *Kosteletzkya virginica* L. [69]. The accumulation of proline under abiotic stress is associated with the plant response to protect against damage to the cellular machinery [70]. In contrast, decreased proline content was also reported in previous studies; for instance, inoculation of Rhizobacteria onto *Arachis hypogaea* L. resulted in a low plant proline content under 100 mM salt stress [71]. In our study, leaf proline accumulation declined in the treatments with Th4 and Th6 in the presence of salt stress. The decreased proline level is unlikely to be a direct downregulation by microbes since it is perceived that abundant proline accumulation in the cytoplasm is one strategy employed by plants to cope with environmental stresses [26]. One plausible explanation for this phenomenon is that microbes induced a decrease in osmotic stress to plants through some yet unknown mechanisms, which in turn induced a downregulation of proline production in plants. It should also be noted that other factors could also influence the level of proline in plants since proline also plays roles other than counteracting the effects of osmotic stress [72]. This suggests that the mechanisms underlying the decreased proline content in the fungal treatments under salt stress are complex and need further investigation.

Salt stress restricts plant biomass accumulation by causing irreversible damage to the photosynthetic machinery [73], which in turn affects photosynthetic gas exchange parameters. However, the application of PGPM counteracts the deleterious effect of salt stress [74]. Likewise, certain *Trichoderma* species are known to enhance photosynthetic efficiency under salt stress [75]. Our results also demonstrated that the net photosynthesis rate of plants grown from noncoated (control) and *Bacillus*-coated seeds decreased under salt stress, but plants grown from seeds coated with *Trichoderma* isolates showed substantial biomass improvement in this regard. This finding is also consistent with a previous report [76] in which the colonization of *Trichoderma virens* improved the photosynthetic rate. The improvement in *Trichoderma*-colonized plants is potentially explained by the stimulation of carbon sink activity through root exudates, therefore manipulating the photosynthesis rate in the leaves [75,76]. Our results revealed that the photosynthesis rates in plants grown from seeds coated with *Bacillus* were unambiguously decreased under salt stress exposure. However, [77,78] claimed that the inoculation of *Bacillus* strains onto pepper and radish improved net photosynthesis under salt stress. Coupled with the improvement of net photosynthesis, the findings of the current experiment also revealed that wheat grown from seeds coated with *Trichoderma* had lower stomatal conductance, intercellular CO_2_, transpiration, and higher water use efficiency under salt stress. Moreover, our results showed that WUE was reduced under salt stress treatment with the uncoated treatments of both cultivars. However, plants grown from seeds coated with *Trichoderma* isolates showed significantly elevated WUE, suggesting that the fungal isolates dramatically improved water use efficiency compared to the bacterial isolate and control. Although the mechanisms were not investigated in this study, recent findings reported the effect of *Trichoderma* inoculation onto *Arabidopsis* and shed some light on the regulation of stomatal aperture and transpiration [79], thereby reducing water loss and improving the regulation of water use efficiency.

Proline is primarily synthesized in the photosynthetic apparatus, and the highest accumulation was found in the chlorophyll of leaves [80]. The optimum degree of proline accumulation appears to be an essential response to a variety of abiotic stresses [81]. In this study, proline synthesis increased with salt stress in both cultivars, although the level of accumulation varied. A positive association was detected between proline accumulation and photosynthetic parameters in *Trichoderma* treatments under salt stress (Table 2 and Figure 2). The photosynthetic performance of wheat plants in the control and *Bacillus* treatments was low under salt stress, even though a high concentration of proline accumulated in the leaves of these plants. Although extensive research has been performed regarding proline metabolism in plants, further study is imperative to explore the discrepancies and metabolic implications of plant-microbe interactions.

Furthermore, the formulations containing *Trichoderma* conidia enhanced crop growth and abiotic stress tolerance as reviewed previously [82]. As mentioned above, *Trichoderma* seed coating strongly influenced proline contents, improved net photosynthesis and water use efficiency, and improved plant biomass under salt stress. This profound benefit suggests the possibility that the studied *Trichoderma* isolates could be considered as a plant biostimulant for amelioration of wheat seedling growth in saline soil. Previous studies reported that formulations containing certain *Trichoderma* species increased abiotic stress tolerance [83], increased nutrient uptake and boosted stress response [82], improved the nutrient uptake and yield of leafy vegetables [84], plant physiology and yield [85], and is regarded as a plant biostimulant.

## 5. Conclusions

Two fungal strains, Th4 and Th6, and one bacterial strain, C7, were isolated from saline soil and pig manure, respectively. Phylogenetic analyses showed that they are closely related to *Trichoderma yunnanense*, *Trichoderma afroharzianum*, and *Bacillus licheniformis*. The three isolates all demonstrated the ability to produce IAA under salt-stressed conditions. Microbial seed coating was performed with salt-sensitive (Shimai) and salt-tolerant (Xiaoyan60) wheat cultivars. The leaf proline content dramatically increased under salt stress for both cultivars. Under salt stress, Th4 and Th6- treated plants showed lower leaf proline contents compared with the uncoated treatments for both cultivars. *Trichoderma* treatments substantially improved the net photosynthetic rate, water use efficiency, and plant biomass under salt-stressed conditions. 

## Figures and Tables

**Table 1:** Indole-3-acetic acid (IAA) secretion (µg mL^−1^) potential of C7, Th4, and Th6 isolates under nonstress (0 mM) and salt stress (200 mM).

Isolates	Salt stress (NaCl)	
	0 mM	200 mM
C7	1.03 ± 0.02 b	1.18 ± 0.02 c
Th4	0.11 ± 0.020 a	0.11 ± 0.02 a
Th6	0.09 ± 0.02 a	0.05 ± 0.02 a

The values are the means of three replicates ± standard error (SE) (*n* = 3). Different letters denote significant differences between treatments after Tukey-adjusted comparisons (*p* < 0.05).

**Table 2:** Leaf proline contents (µmol proline g^−1^ FW) of wheat cultivars (Shimai and Xiaoyan60) under microbial seed treatment and salt stress.

A			
Cultivars	Seed Treatment	µmol Proline g^−1^ FW	
		0 mM NaCl	200 mM NaCl
Shimai	Uncoated	2.16 ± 1.24 a	67.98 ± 1.24 e
	C7	0.97 ± 1.24 a	56.38 ± 1.24 d
	Th4	2.67 ± 1.24 a	59.23 ± 1.24 d
	Th6	1.59 ± 1.24 a	61.09 ± 1.24 d
Xiaoyan60	Uncoated	12.76 ±1.24 b	28.07 ± 1.24 c
	C7	0.55 ±1.24 a	69.18 ± 1.24 e
	Th4	1.50 ± 1.24 a	17.20 ± 1.24 b
	Th6	1.28 ±1.24 a	3.50 ± 1.24 a
**B**			
**Treatments**		***F***	
C		560.93 ***	
M		122.04 ***	
S		4666.77 ***	
C × M		149.81 ***	
C × S		739.18 ***	
M × S		122.49 ***	
C × M × S		168.61 **	

(**A**) The values are the means of six replicates ± SE (*n* = 6). Different letters denote a significant difference between treatments after Tukey’s HSD test (*p* < 0.05). (**B**) Three factorial ANOVA results for proline content in the two wheat cultivars (C) under microbial treatments (M), salt stress (S), and their interaction (C × M, C × S, M × S and C × M × S). ** and *** denotes that the mean difference is significant at the *p* < 0.01, and *p* < 0.001 levels, respectively.

**Table 3:** Photosynthetic parameters of two wheat cultivars (C) in response to microbial treatment (M), salt stress (S), and their interaction.

Treatment	Photosynthetic Parameters				
	*A*	*g_s_*	*C_i_*	*E*	*WUE*
C	0.60 *ns*	2.64 *ns*	0.00 *ns*	1.67 *ns*	10.43 **
M	25.29 ***	2.65 *ns*	18.69 ***	6.53 **	73.30 ***
S	258.58 ***	117.77 ***	0.00 *ns*	138.18 ***	9.66 **
C × M	8.36 ***	1.67 *ns*	3.54 *	0.39 *ns*	1.97 *ns*
C × S	4.42 *	2.90 *ns*	1.46 *ns*	2.14 *ns*	1.23 *ns*
M × S	13.63 ***	2.92 *	8.17 ***	6.41**	22.59 ***
C × M × S	2.18 *ns*	2.04 *ns*	1.39 *ns*	0.78 *ns*	2.42 *ns*

The values in the table represent three factorial ANOVA results for the photosynthetic parameters: net photosynthesis (*A*), stomatal conductance (*g_s_*), intercellular CO_2_ (*C_i_*), transpiration (*E*), and water use efficiency (*WUE*) under microbial treatment (M), salt stress (S), cultivar (C) and their interaction (C × M, C × S, M × S and C × M × S). *, **, ***, and *ns* denotes that the mean difference is significant at the *p* < 0.05, *p* < 0.01, *p* < 0.001 and non-significant levels, respectively.

**Table 4:** Plant biomass production for the two wheat cultivars (C) in response to microbial treatment (M) and salt stress (S).

Treatments	*F*	
	Shoot	Root
C	50.391 ***	228.01 ***
M	69.13 ***	65.31 ***
S	666.32 ***	846.81 ***
C × M	6.40 **	5.47 **
C × S	11.35 **	141.61 ***
M × S	1.33 *ns*	3.47 *
C × M × S	7.05 ***	14.43 ***

The values in the table represent analysis of variance (ANOVA) test for biomass of the wheat cultivars (C) under microbial treatment (M), salt stress (S), and their interaction (C × M, C × S, M × S and C × M × S). *, **, ***, and *ns* denotes that the mean difference is significant at the *p* < 0.05, *p* < 0.01, *p* < 0.001 and nonsignificant levels, respectively.
